# Sensor Actuator Network for In Situ Studies of Antarctic Plants Physiology †

**DOI:** 10.3390/s22228944

**Published:** 2022-11-18

**Authors:** Krzysztof Herman, Mauricio Montanares, Leon Bravo, Joanna Plenzler

**Affiliations:** 1 Department of Electrical and Electronics Engineering, University of the Bío-Bío, Concepción 4051381, Chile; 2 Department of Electrical Engineering, University of Concepción, Concepción 4070386, Chile; 3 Soil Interaction and Natural Resources Biotechnology, Scientific and Technological Bioresource Nucleus, University of the Frontier, Temuco 4811230, Chile; 4 Institute of Biochemistry and Biophysics, Polish Academy of Sciences, 02-106 Warsaw, Poland

**Keywords:** sensor networks, thermal actuators, antarctic plans, IoT

## Abstract

This article documents a custom sensor–actuator network designed and implemented as a part of experimental setup, where a long-term phenological response of antarctic plants is studied. The first part of our work presents the context of the study, reports experimental methods used in antarctic plant field studies, and characterizes the environmental conditions and logistics facilities available on the measurement spot. After contextualization of the research, we present, in detail, both the network itself and some results obtained during the Antarctic summer seasons between 2019 and 2022 on the King George Island, South Shetlands. The results collected with our network and correlated with selected data registered with a reference automatic meteorological station reveal the thermal plants response. The groups of plants individuals, which were actively warmed using thermal actuators, show the nighttime temperature difference, in reference to the air temperature, of 5 ${}^{\circ }$C, which complements the daytime difference caused by the passive method of open top chamber (OTC) used in previous studies carried out in the same localization.

## 1. Introduction

The ongoing climate change is a fact; however, it is not uniform across the planet, revealing that the extremes are situated on the poles of the planet. According to the long-term meteorological data reported in the paper [1], the region of the Antartic Peninsula has warmed around 3.7 ${}^{\circ }$C during the last 100 years and this rate of change, 0.03 ${}^{\circ }$C/year, has been four times faster than the warming rate of the rest of the globe. Each day, the “breaking” news, such as that reported in the New York Times on 6 February [2] “… a new air temperature record was registered in the Argentinean station *Esperanza* in the Antarctic”, thrills the public less. A few days later, some other research stations have confirmed the high value of the air temperature in the region of the Antarctic Peninsula. The above-mentioned facts mean that the polar and subpolar regions are a research subject of many scientists investigating climate change and its direct and indirect impacts on the environment. Since the climate affects both: biotic and abiotic parameters of ecosystems, not only direct climate-related physical variables are being studied, but also a variety of indicators and phenomenons related to the fauna, flora, glaciers, marine ecosystem, geology, geomorphology, atmosphere and many more. The presence of plants and their ability to withstand the extreme environmental conditions in these remote areas have been investigated by various research groups. The region of Arctic is much more rich and diverse in terms of vascular native plants comparing it to the Antarctic remote areas, where only two vascular plants species were able to colonize some of the most southern regions of the planet. The vascular plants in the Antarctic are represented by the following ones: the antarctic hair grass *Deschampsia antarctica DA* and the antarctic pearlwort *Colobantus quitensis CQ*, which have been studied for more than a century, as it was reported in the article [3]. There are plenty of articles [4,5,6] that reveal how the temperature changes impact the plants, their growth, reproduction capabilities and internal processes such metabolism and photosynthesis. In 1994, in the article [7], the plants were recognized as bioindicators (more precisely its expansion rate) of climate change due to: (1) relatively rapid response to the climatic changes in comparison to most of the other terrestrial macro-organisms, and (2) the strong correlation between the increase in number of individuals and populations, and a warming trend in summer air temperatures. According to the articles [8,9], the major environmental factors, which impact the plant’s physiology, and therefore its presence in a particular zone, are the following: low summer temperature, water availability, length of the growing seasons and soil availability. Due to climate change, temperature changes, retreating glaciers and other propitious factors increasing in the surface covered by flowering plants, the number of populations and the increase in plant size have been observed during the last 20 years.

**Figure 1:**
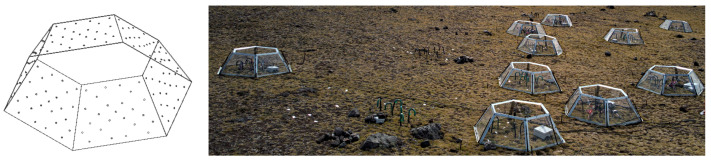
Visualization of an OTC chamber used in the experiments in order to simulate the day time warming and a photograph of a measurement spot (King George Island, South Shetlands), with OTC installed.

In situ measurements, biological samples collection and analysis represent retrospective methods of reasoning, where conclusions are based on historical data outcomes and predictions are made using models developed using the same data. What if we could alter the growth conditions of the plants and measure their response? If the global trend of warming will continue, what would be the response of the plants? Those are valid questions. There are plenty of studies where the antarctic plants were grown in a laboratory under strictly controlled conditions; however, performing the same in situ, especially in the Antarctic, sounds challenging. One of the methods used in order to locally increase the environmental temperature, thus the plant’s temperature, is a passive method of OTC, *Open Top Chamber*. The OTC, visualized in Figure 1, is a hexagonal prism made of transparent acrylic, where the base of the prism is around 1 square meter.

The ventilation perforations ensure that the inside temperature does not exceed a few grades of Celsius in reference to the outside temperature. One of the principal issues related to the OTC is that it alters the temperature only during the daytime and the supposition of the continuity of the future warming trend is based on the daily mean temperature understood as a 24 h period. Finally, the rest of the article describes a solution, which addresses this particular problem by means of a sensor actuator network for nocturnal warming in a context of antarctic plants physiology in situ studies.

### 1.1. Review of Methods Used in the Field Studies of Antarctic Plants

In general, the biological studies in the Antarctic are based on the sampling and sample analysis; however, the physical conditions related, for example, with the local meteorological conditions, can be, and in fact are, measured using automatic methods such as automatic weather stations or data loggers. In our particular case, the principal problem to solve is a heat transfer towards the biological tissue of the antarctic plants in its natural habitat in order to maintain plant’s leaves temperature higher than the ambient temperature. The constant temperature difference can be obtained thorough a sensor–actuator network together with a dedicated control system. Since the heat transfer process depends on the source–medium–object characteristics, it is necessary to identify the system thermal parameters in order to correctly design the whole system. Although there are some studies that reveal thermal properties of some common plants described in the article [10] and methods to estimate it reported in the following study [11], there are no records of similar data in the case of the antarctic plants. There are two supposed reasons for that: (1) antarctic plants are not in the mainstream of the plant’s biology in opposition to the agriculture and food processing, (2) antarctic plants because of their small dimensions present serious problems during measurements (the instrumentation equipment is sized to measure bigger objects). An overview of methods for leaves temperature measurements was presented in the paper [12]; however, all the measured objects (leaves) were sized, at least, an order of magnitude higher in surface in comparison to the antarctic plants. In the field studies, the most common method of measuring the antarctic plants temperature is the application of small thermocouples and IR sensors. At the moment, there is one published article that reports active heat transfer in field experiments using IR radiators [13].

**Figure 2:**
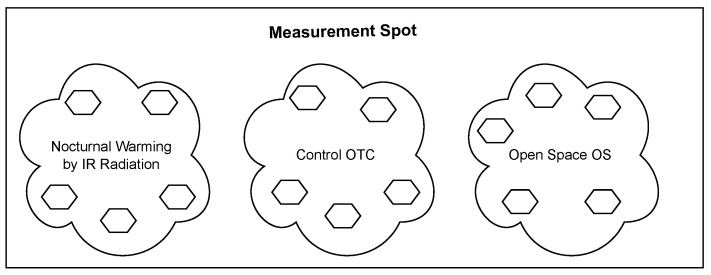
The main diagram of the experimental setup organized by three main groups. Each group contains five OTC, where six plants are monitored (three DA and three CQ).

**Figure 3:**
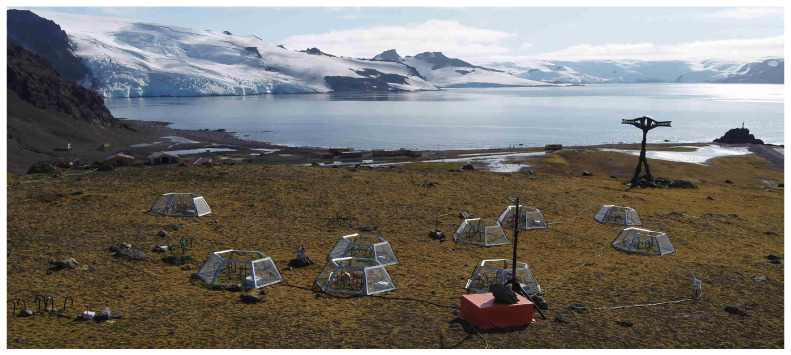
The aerial view of the measurement spot. In the foreground, the sensor actuator network is presented. In the background, H. Arctowski Polish Antarctic Station and the Admiralty Bay. A schematic, top view of the spot is presented in Figure 5. A totem on the right is a W. Puchalski grave, which is listed as an HSM-51 (Historic Site or Monument) by the Antarctic Treaty System.

The presented system was designed to be applied in field experiments in order to study the antarctic plants and to validate a biological hypothesis: *Antarctic vascular plant morpho-physiological traits are more responsive to the increase in minimal nocturnal temperatures rather than the increase in daylight average temperature,* where a methodology based on statistical methods was proposed. The methods involve a variance comparison between groups known as ANOVA test, where the experimental data, associated with the groups being compared, are processed. In terms of the proposed system, the groups are represented by different measurement spots, where the plant’s leaves temperature is monitored. The experiment assumes three different groups: (1) an active warming group, where heating elements are installed inside the OTC’s (denominated as Nocturnal Warming by IR radiation on the Figure 2, (2) a group with OTCs without heat actuators (denominated as Control OTC on the Figure 2), and (3) a group without OTC often called a control group (denominated as Open Space on the Figure 2). The ANOVA method is founded on a Gaussian distribution of the measured variables, where the condition of a sufficient number of samples has to be satisfied. The above requirements set up each group to be represented by various measurement points, under the assumption of homogeneous measurement conditions within these groups. In our particular case, the measurement methodology has considered measurements of three individuals per spot per species. In other words, we had to scale prepare the system to be able to monitor six individual plants per OTC (or open space). In total, we had to manage measurements of the leaves temperatures of 90 individual plants and actively heat 30 of them. Due to the spatial distribution of the plants in the field and temperature measurement methods (local sensing), the system should be distributed as conforming to a scalable sensor network.

### 1.2. Detailed Characterization of the Measurement Spot

The field measurement campaign was carried during the Antarctic summer periods in the years 2019–2022 in the vicinity of the Polish Antarctic Station “Arctowski” ($62^{\circ }09^{\prime }34^{\prime \prime }$ S $58^{\circ }28^{\prime }1^{\prime \prime }$ W), King George Island. The station is operating all year round and provides access to the 220 V power grid and low speed internet satellite connection up to 1 Mbit/s. The measurement spot is situated approximately 500 m away from the station and elevated around 60 m above the sea level (a.s.l), with a clear line of sight between the spot and the station shown in Figure 3.

As mentioned in the article [14], on the spot, the respective densities of *C. quitensis CQ* and *D. antarctica DA* are in the range of 70–200 and 20–150 individuals m${}^{-2}$. The plants are small in size, the average values of the surface area and the height for *C. quitensis* are 2 cm${}^{-2}$ and 2.5 cm, respectively, and the corresponding values for *D. Antarctica* are 10 cm${}^{-2}$ and 4 cm.

According to the recent meteorological data from the investigated area for the period 2013–2017 [15], the summer (December–March) mean monthly air temperature varies between 0.3 ${}^{\circ }$C in December (the coldest summer month) and 1.6 ${}^{\circ }$C in February (the warmest summer month). During summer, mean daily air temperature is predominantly higher than 0 ${}^{\circ }$C (on average, from 58% of days in December to 76% of days in February). The annual mean relative humidity was 78.1% [15]. The Antarctic region is famous for its strong winds. The mean monthly wind speed at Arctowski Station during the period 2013–2017 in summer was from 4.6 m/s (Dec.) to 5.5 m/s (Mar.), and the mean number of days when the maximum wind speed exceed 20 m/s was reported from 3.0 (Dec.) to 11.6 (Mar.). The predominant wind direction was SW (31%). The monthly sum of precipitation in the summer period (2016–2017) was reported to be in the range of 14.6–170.1 mm [15]. Continuous snow cover rarely occurred during summer. During summer in 2016–2017, there were 0–5 days with snow cover per month [15], and during the investigated period (summer 2019/20 and 2020/21), only 0–2 days with snow cover per month (personal observations).

### 1.3. Selected Recent Advances in Sensors and Sensors Technology in the Regions of the Arctic and Antarctic

Although the title of the subsection could be the title of a separate article, in this paragraph we would like to present some reviews of sensors and technological solutions, which were applied in various research studies in the polar regions during the last decade, in fact during the last 5 years. Since the polar regions are the main reservoir of freshwater in the form of snow and ice, there are plenty of studies related to this matter, where new technological solutions are emerging. The standard methods based on image processing and matching [16] are being constantly modified due to a proliferation of image capture techniques (satellite imaging, multispectral cameras, stereoscopy, UAV HD imaging). Apart the snow (ice) cover area, some more parameters are measured in order to estimate, for example, glacier mass balance and its impact on the local weather conditions. Those are: snow depth and snow water equivalent (SWE). Some new methods of evaluation of these parameters were recently reported: (1) a microwave resonator tested in Concordia station and described in [17], (2) the article [18] reports a 24 GHz radar for snow depth, ice thickness and SWE evaluation, and (3) ground-based snow depth and density estimation methods using ultrasonic waves tested on King George Island and reported in [19]. It is rather straightforward that the snow and ice properties depend on the meteorological conditions, which are being measured using various technologies, each day more automated and autonomous. Automatic weather stations and data loggers are commonly used in the polar regions; however, due to the absence of power sources and limited availability of telecommunications services, some alternative technologies have emerged. Some wireless sensor networks, based on Wi-Fi [20] and LoRa [21], are proposed in order to: (1) acquire information about spatial distribution of measured physical variables (mostly meteorological data), (2) minimize the data delivery time, and (3) provide feedback about the sensor failure. Apart from the Irydium-based satellite service, there were some attempts to provide reliable data transmission from the sensors or sensor networks to an online database, among them a long haul radio link from the Greenwitch Island to Spain [22,23]. Nevertheless, at the moment the article was being written (Sep 2022), SpaceX together with National Science Foundation have announced [24] that the Starlink service at the McMurdo station was successfully launched. Some advanced algorithms, discrimination [25] and machine learning [26], were recently reported, which support the global tendency related to so-called smart sensors, which process the raw data on site and a deliverable in the information about the process being monitored, for example the local weather forecast.

## 2. Nocturnal Warming System—Detailed Description

**Figure 4:**
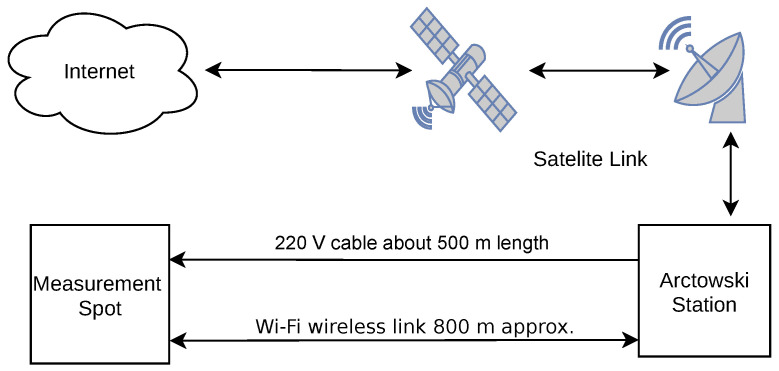
A global diagram of the custom network insertion into the Arctowski station infrastructure.

In order to fulfill the requirements commented on in the previous sections, the experimental setup has to be organized as a distributed sensor actuator network, mainly due to the spatial plant distribution and methodology used. The system is going to be exposed to the harsh condition because of the high value of the relative humidity, possible precipitation (rain and snow) and wind blasts. Apparently, near-0 ${}^{\circ }$C temperatures do not cause problems themselves; however, in conjunction with the high humidity and freezing–defreezing cycles, it can affect the system’s mechanical components, especially the sealing elements. Since the detailed description of the control methods applied for this project is reported in [27], this part will not be mentioned in this section. The overall system diagram is presented in Figure 4. The three spots shown in Figure 2 represent three groups to be compared. Each group consists of five OTC, where the plant’s leaves temperature, air temperature and air relative humidity are monitored periodically. The system is able to: (1) monitor the leaves temperature of 90 plants, (2) measure air and air humidity at each node, (3) measure status information such as supply voltage level, error status, and core temperature at each node, (4) backup and report the collected information, and (5) perform active plant heating during the nighttime. Since the experiments involve actuation using heat sources, the 220 V power line was delivered to the spot. Availability of the AC power gave the opportunity to supply power to all modules using locally installed DC sources. Moreover, all the modules were connected using a copper wire in order to: (1) power up all the modules, and (2) implement a reliable communication system. The three groups were distributed spatially within the radii of <50 m, which constrains the communication system, which has to collect the data from each node and forward them to the gateway and then to the cloud. The data forwarding can be done using the two methods: (1) satellite data transfer via “Arctowski” and the station’s local LAN or (2) using the GSM data link provided by the Brazilian station. The above scenario suggested incorporation of some additional nodes being able to maintain the communication, sensor data transfers and other services such as local backup, time synchronization, network monitoring, node management, and event scheduling.

During the design stage, we have considered that the cable network on the spot should be able to transfer data at a relatively low bit rate, and the network reliability should be the principal factor to evaluate. There are plenty of variations of Ethernet-based LANs; however, the high throughput and additional hardware needed (switches, cables, interfaces) seem to be inadequate for our network, considering them as possible single points of failure (SPOF). Some industry standards such as CAN *(Control Area Network)* and MODBUS (a data communications protocol) were revised, and finally the CAN protocol [28] was selected and applied. The CAN network offers a complete protocol solution beginning from the electrical level up to the data encapsulation within frame with CRC (Cyclic Redundancy Check) correction and error reporting. Moreover, CAN bus offers differential signaling together with a hardware-based negotiation that gives the possibility to organize the network in a single bus topology, which reduces the number of connections.

**Figure 5:**
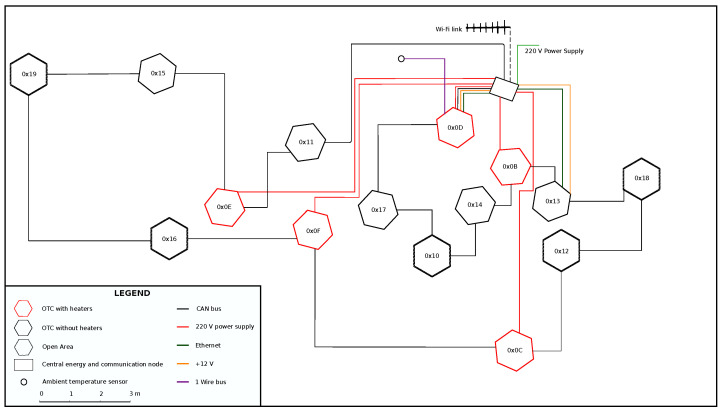
A schematic view of the measurement setup installed in Antarctica.

In Figure 5, the spatial distribution of the nodes is shown.

The figure, apart from the nodes aggregated in groups, shows respective interconnections and the center rectangular point, where: (1) 220 power is divided and distributed to the heating nodes, (2) security devices are installed (fuses, local ground point), (3) DC power supply, DC backup power supply and 12 batteries are installed in order to provide 12 V system voltage to each node, and (4) wireless is installed in order to communicate gateways with the Arctowski station and thus, with internet.

**Figure 6:**
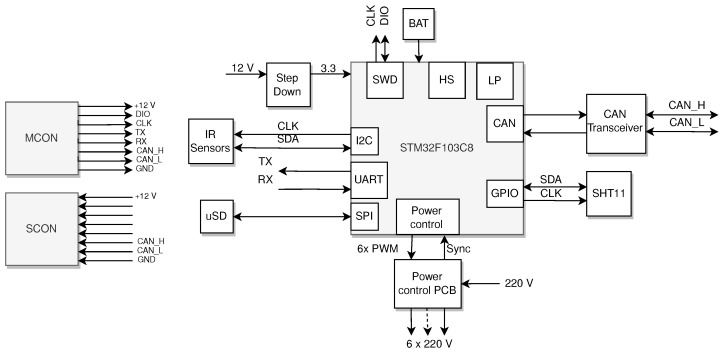
A schematic diagram of the ARM-Node. Description in text.

The basic network unit, designed to handle the data acquisition and temperature control, was a module named ARM-Node (after the ARM Cortex M processor being the center of the unit). The mentioned ARM Cortex M3 processor was selected due to the rich communication peripherals such as CAN, I2C (Inter Integrated Communication) also known as SMBus or Two Wire interface, SPI (Serial Peripheral Interface), UART (Universal Asynchronous Receiver Transmitter); advanced timers with PWM channels; embedded RTC clock with backup domain and a variety of low power consumption modes. As it is shown in Figure 6, the ARM-node is powered by a 12 volt power supply line voltage distributed together with the CAN network using an ethernet cat6 cable. The processor was clocked using two crystals at 8 MHz and 32 kHz, respectively, which permits us to switch between low/high power modes and to preserve (together with the backup battery) the backup domain registers, and thus the time, date and critical data during reset.

**Figure 7:**
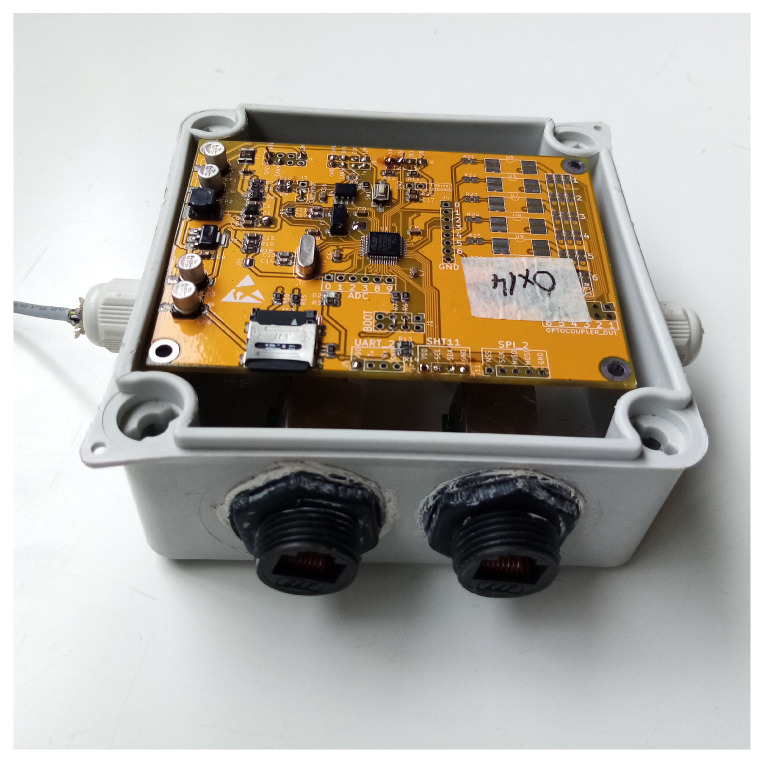
The ARM-Node inside IP67 enclosure. The rugged RJ45 connectors and cable glands are show.

The node has its own local data backup implemented using a micro SD card interfaced via SPI module that, in the case of the network failure, provides a mechanism of local data backup and continuity of operation. The device can be accessed via CAN network and throughout the UART interface in a debug mode. The nodes with thermal actuators use six PWM independent channels in synchronized to the power line voltage zero crossing in order to generate trigger pulses for independent, optocoupled triacs. The node was encapsulated in a plastic enclosure with IP67 protection grade. The network connection was realized using two rugged RJ45 connectors, while the sensors were connected using four wire cables and silicon sealed cable glands (Figure 7). The power supply for the ARM node network was designed to be 12 V stepped down by a node converter to be 3.3 V at the node. This solution permits us to distribute the power supply and implement CAN bus using CAT 5,6, AWG 26 cable and guarantees protection against the voltage IR drops at the end of the bus. The basic feature of the network node was to perform the plants leaves temperature measurements and to implement the control mechanism in order to drive heat sources on the plants, where the nocturnal warming was required. The contactless, automotive grade, temperature sensor MLX90614 was chosen to monitor the plant temperature. Six MLX sensors, organized in a daisy chain, are connected to the node, where the sensors–node communication was implemented using SMBus protocol. The control of the heat transferred to each of the six plants within one OTC was implemented using PWM modulation, where the heat sources (25 W ceramic heaters) were controlled independently, thus each plant temperature was controlled separately.

**Figure 8:**
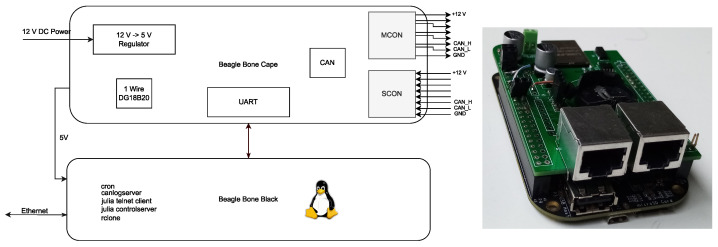
The block diagram of a system gateway based on the Beagle Bone Black with the custom cape.

In our case, the network consisted of 15 ARM-Nodes, which collected data from over 120 sensors and heated up 30 plants individually. In order to manage the local CAN-based sensor network and make a bridge between CAN and the station’s LAN network, two single board computer modules were incorporated. The Beagle Bone Black based on AM335x ARM Cortex-A8 Sitara processor were utilized as hardware platforms. The computer’s hardware involves 100 MBit Ethernet connection, CAN 2.0 module, GPIO, UART, RTC, 1-Wire, USB 2.0. In opposition to the ARM-Node, where all the applications are bare metal, interrupt-driven applications, the Beagle Bone node runs Linux Debian OS, which permits it to exploit the mechanisms of the OS. The principal goals of the Beagle Bone are: (1) to schedule the measurements, (2) to collect and store the biological and system data, (3) to manage a local database, (4) to synchronize the database with the cloud, (5) to manage the sensor network via remote connection, (6) to report system status, and (7) to measure a reference temperature using a DS18B20 temperature sensor and distribute it to the local controllers. Although the Beagle Bone Black does have the CAN 2.0 support, the CAN level transceiver had to be installed together with additional 5 V voltage regulators to source current to the GSM (Global System for Mobile Communications) module. The above was realized in the form of a cape for the BBB module, see Figure 8.

Two Beagle Bone Black modules were connected via wireless router to the station Wi-Fi in order to gain access to the satellite link. In the get remote access to the BBB module, a Zerotier [29] network client was installed, thus one can access the remote module using, for example, the SSH protocol. The measurement automation operates in the Linux user space, taking advantage of Linux built-in applications. The CAN network is managed by the can-utils package [30], where one can send and register the CAN frames. To perform the periodical tasks, a cron mechanism was extensively used, especially to implement the main application, which was based on Julia language. We have implemented an asynchronous control server that receives cron scheduled triggers related to particular actions, such as: local temperature measurement, CAN bus management, request for data, and data synchronization. In a case of an error, an exception is thrown and reported in the system log. The reception of the CAN frames is based on client–server application as well. We have used the canlogserver from the can-utils package, which exposes the incoming CAN frames as a plain text on a particular TCP port. We have developed a telnet client using Julia language, where the data are not only captured, but also parsed and categorized, due to the fact that the data in the CAN frame are sent in a binary form (not string) using a custom frame format described in [31]. The custom telnet client uses a database based on Julia DataFrames, which dumps the data in the form of CSV files. Once per day, the local data backup was performed and synchronization with a Google Drive was made using rclone tool. In order to explain the network operation, it has to be divided into 2 parts: ARM-Node operation and Beagle Board operation. The ARM-Node after booting recovers its fixed ID from the code, day, and time from the backup domain and, if it corresponds, PID (proportional, integral, differentiate) coefficients of the controller, it also sends a frame over the network that it is ready to operate. Since then, the node is ready to be managed using commands send over the network. If timeout has occurred, (no command arrives) the node perform measurement and stores results in the local backup (SD card). In case of a node with active heating, the node performs heating if the corresponding flag (set by Beagle Bone) was set and recovered from the backup domain. The unit measurement contained: timestamp, successive measurement iterator, power supply voltage, core temperature, six MLX sensors readings (corresponding to six plants), air temperature, and air humidity.

Because of possible failure, we had two Beagle Bone single board computers in the can network, where one was manually set as a master node. During boot, the service of canlogserver was activated. Since then, the Julia custom control server and telnet client could both monitor the network and control the network operation. Each minute, cron sent a control command to the control server in order to trigger a local reference temperature measurement and distribute a call for measurements to each ARM-Node. Once distributed, the ARM nodes responded to the call with four frames that contained the measured data. The frames were captured by booth Beagle Bones individually by respective custom telnet clients and stored in respective data frames. At the end of the day, typically at 20:00 h, the call for warming was distributed along the network, which as a result, switched on heating on each active ARM node, since that moment the reference temperature measured by the master Beagle Bone was broadcast along the network in order to feed PID controllers with a reference values. The nocturnal warming was usually switched off at 6 am. Additionally, once per hour the data from the data frame structure were saved to a respective CSV file, and once per day an update of RTC clock was performed in a whole network.

## 3. Results

The measurement campaigns took place during the summer seasons, 2019–2022. The network was deployed on the spot only for the summer and uninstalled and kept safe during the winter time. The data were collected not only using our sensor network, but also using a reference automatic weather station (AWS), with the sensor presented in the Table 1.

**Figure 9:**
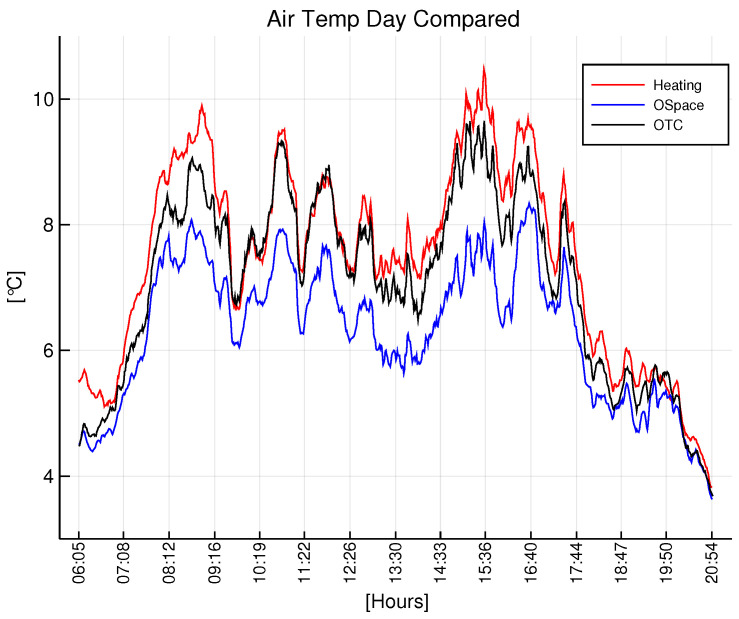
Daytime temperature at nodes of each group.

**Figure 10:**
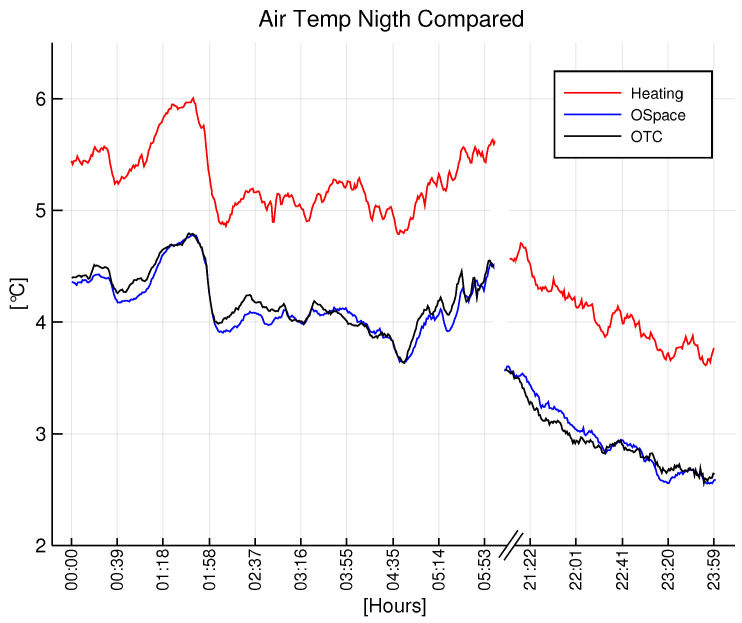
Nighttime temperature at nodes of each group.

In order to render the principal functionality of the system, a daytime Figure 9 and nighttime Figure 10 average temperature of the air of each group are presented.

The data show the substantial difference between the groups. During the daytime, there exists a systematic gap between the open space and nodes inside the OTCs, which supports the greenhouse effect of the OTC. Correspondingly, during the nighttime, the temperature of the open space and control OTC are almost perfectly aligned, which supports the lack of the greenhouse effect inside OTC during the nighttime, and it validates our study.

**Figure 11:**
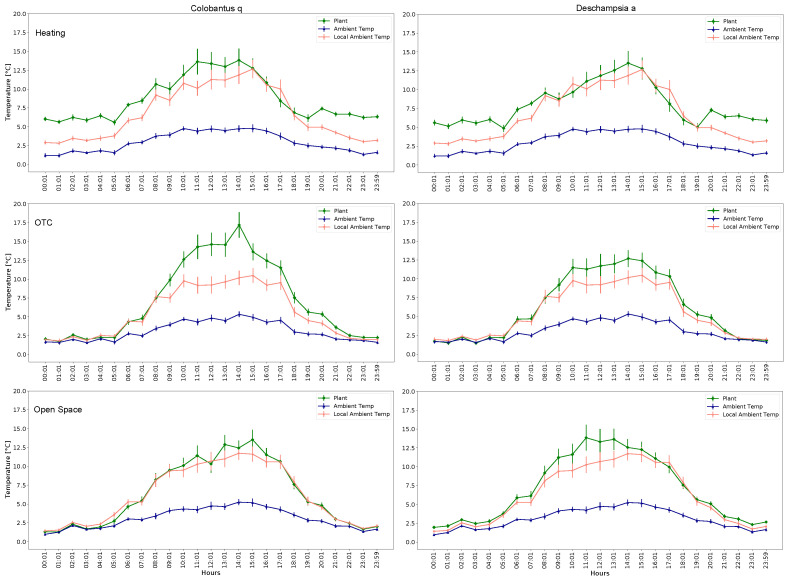
Leaves temperature, air local temperature and reference temperature segmented by plants and groups.

In Figure 11, the mean and standard deviation of the plant’s temperature, local temperature and reference temperature are shown.

**Figure 12:**
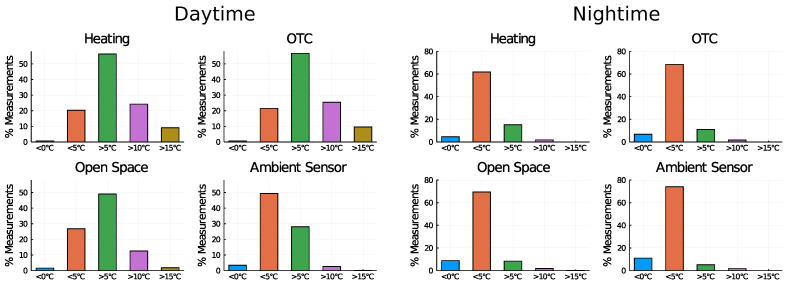
Customized temperature histograms segmented by groups and period (daytime or nighttime).

In Figure 12, a customized histogram of the temperature is shown.

The data were rendered by segments in order to represent the number of “events” such as very cold nights or very warm days, that is useful when correlating with the physiological response of the plants.

**Figure 13:**
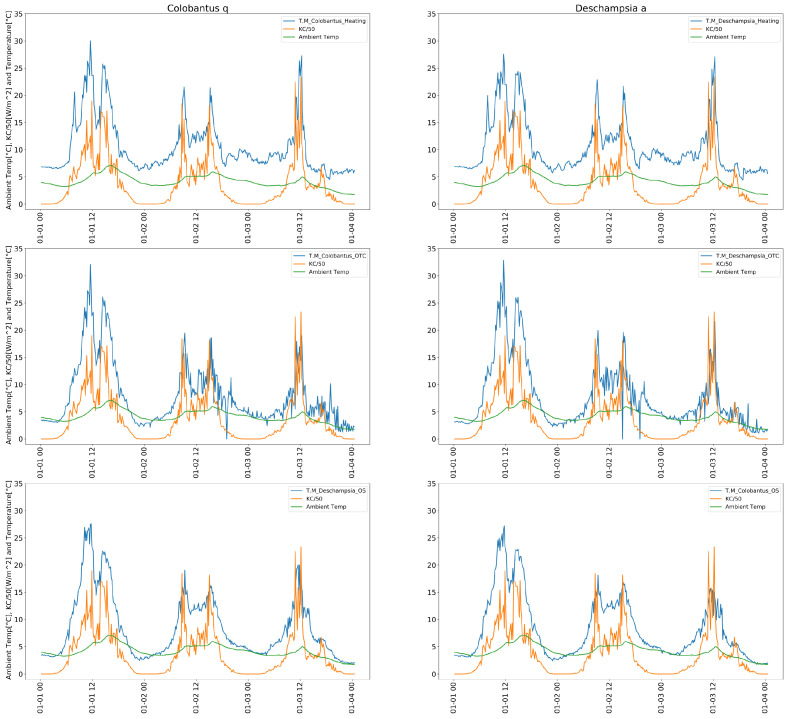
Time series of leaves temperature, segmented by species and group, together with solar radiation and ambient temperature measured by automatic weather station in a period of 1–4 January 2021.

In order to investigate a dependence between leaves temperature, solar radiation and ambient temperature, some selected data related to sunny days were shown on the Figure 13.

**Figure 14:**
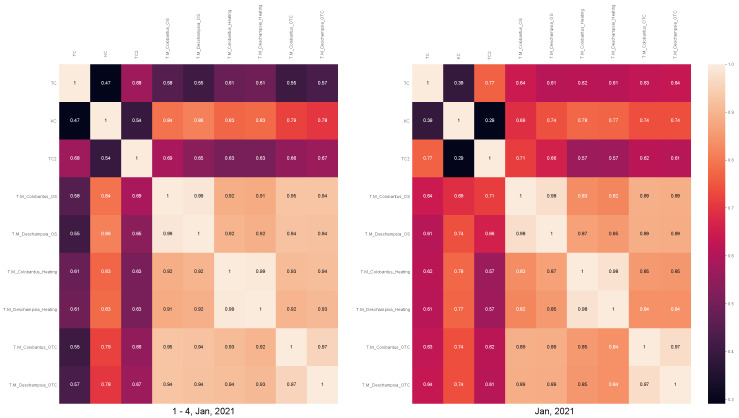
Correlation diagrams between data from weather station (TC—air temperature, TC2—soil temperature, KC—radiation) and leaves of the plants in different groups in a period of 1–4 January 2021 and whole month Jan 2021.

Additionally, correlation plots shown in Figure 14 compare the data presented previously on the Figure 13 taken during the first days of January with data taken during the whole month of January 2021.

## 4. Discussion

The first point of discussion is that it is very difficult to compare our solution to anything, at least similar. The only one existing study carried out in the vicinity of the Palmer Station in the region of the Antarctic Peninsula, reported in the article [13], used fixed power IR heaters mounted over experimental traits of plants. Although, the experiment was carried out in the Antarctic the plants were transplanted into pots and finally the growth conditions were altered. Some interesting insights can be found analyzing data reported in the article [32], where some relationships between radiation, leaves temperature and air temperature were shown. Considering two methods of a leaf temperature measurement: (1) direct, as reported in [32], and (2) non-contact using infrared radiation, we can argue if the first method is adequate in the case of measurements of such small objects as the leaves of the antarctic plants. The above can be justified using two arguments: (1) the specific heat capacity of the thermocouple and the leaves, and (2) the thermal coupling between leaves and the thermocouple. A typical copper–nickel thermocouple wire has a specific heat of 393 J/kg/K${}^{-1}$, while the same parameter of some leaves of plants reported in [10] can have a value in a range of 255–2267 J/kg/K${}^{-1}$ for fresh leaves. Assuming ideal thermal coupling between the leaf surface and the thermocouple still there is a substantial systematic offset in the results of measurements introduced by the thermocouple. The method presented in our article estimates the leaves temperature using a contact-less method as a spatial integral of infrared radiation measured by the MLX90614 sensor. The sensor itself guarantees the accuracy of $0.2{\;}^{\circ }$C

Analyzing the results presented in Figure 9, Figure 10, Figure 11 and Figure 12, it becomes clear that the OTC and the active warming system alternate the microclimatic conditions inside the OTC with the nocturnal warming system installed. However, a careful distinction should be made between air temperature and leaves temperature. Since the infrared heaters radiate heat on individual plants, the effect of heating and maintaining the $5{\;}^{\circ }$C difference is evident (as a result of high performance of individual PID controllers). Nevertheless, the distinguishable difference in air temperature inside the OTCs with a nocturnal warming system is also evident, although it is a side effect of dissipated heat.

The data from the weather station shown in Figure 13 and correlation plots shown in Figure 14 provide us with some interesting insights about the experiment. A strong correlation between plant leaves temperature and solar radiation can be observed, especially in the selected period of 1–4 January 2021. The air temperature does not respond immediately and there exists a lag between the peak of radiation and the peak of temperature (in a simplified model it would be related to the dynamics of a heat transfer). We can also observe a very strong correlation within the corresponding groups (Heating, OTC, and Open Space) and moderate correlation between air temperature and leaf temperature.

## 5. Conclusions

In the article, a sensor—actuator network for in situ studies of antarctic plants physiology was demonstrated. Although, until the moment, we cannot provide any data related with plants physiology, which are being under analysis, we can state that our solution has accomplished its role by alternating local microclimatic conditions of the plant’s growth. We have observed both: (1) the increase of the leaves temperature and (2) the increase of ambient temperature inside the OTCs, where an active heating system was installed. One of our final remarks is that the plants’ leaves temperature is strictly correlated with the solar radiation and can reach temperatures much higher than the ambient temperature. Additionally, it seems that the introduced non-contact method of temperature measurement is more confident in terms of systematic error introduced by the measurement method. The final remark we would like to share is that the presented study is unique and distinctive because it explores methods, where an active component is introduced into the local environment. Traditionally, and due to environmental restrictions, any introduction of biological or non-biological material is prohibited or limited in the Antarctic. From this point of view, the alteration of the microclimatic growth condition of the antarctic plants using state of an art technology surely opens new research opportunities. 

## Figures and Tables

**Table 1:** Reference weather station sensors.

Equipment Function	Type
Air temperature sensor at 2 m	Vaisala HMP155
Air temperature sensor at 0 m	Campbell 107
Solar radiation sensor	Apogee CS300
Data logger	Campbell CR1000

## Data Availability

Not aplicable.
